# Synthesis of NiO:V_2_O_5_ nanocomposite and its photocatalytic efficiency for methyl orange degradation

**DOI:** 10.1016/j.heliyon.2018.e00581

**Published:** 2018-03-17

**Authors:** Salam A. Mohammed, Lamya Al Amouri, Emad Yousif, Ali Abd Ali, Fazal Mabood, Hazim F. Abbas, Sausan Alyaqoobi

**Affiliations:** a Department of Chemical and Petrochemical Engineering, College of Engineering and Architecture, University of Nizwa, 616, Nizwa, Oman; b Department of Chemistry, College of Science, Al-Nahrain University, Baghdad, Iraq; c Department of Chemistry, College of Science, University of Nizwa, 616, Nizwa, Oman; d DARIS Centre for Scientific Research and Technology Development, University of Nizwa, Oman

**Keywords:** Materials chemistry, Physical chemistry, Chemical engineering, Inorganic chemistry

## Abstract

Vanadium oxide has been largely exploited as a catalyst in many industrial applications. In this article, we show the synthesis of vanadium oxide (V_2_O_5_): Nickel Oxide (NiO) composite using sol-gel method at optimum conditions. The composite nanomaterials were used to remove methyl orange from waste water via harnessing the photocatalytic activity of it which showed an excellent efficiency of removal at 88%, and 93% after the exposure to the light, and light with heating respectively. This will pave the way into further implementation of these nanomaterials in the removal of some other dyes and contaminants from wastewater.

## 1 Introduction

Organic substances are usually considered as pollutants in wastewater from industrial plants and they must be removed prior to discharging them the environment. Dyes and pigments are widely used in several industries such as production of fabrics, food. However, the accidental disposal of these dyes into wastewater has caused a severe environmental and health problems. Therefore, it is necessary to develop universal methods to remove dyes from wastewater [1, 2, 3, 4]. In the past decade, photocatalysis has been widely used across the globe as a robust technique to remove contaminants such as dyes from waste [5, 6, 7] as catalytic reaction involves the production of the catalyst by absorption of light [8]. A plethora of reports have demonstrated that hazardous organic compounds such as dyes can be decomposed using photo charge carriers originated from metal oxide semiconducting nanomaterials [8, 9, 10, 11, 12, 13, 14]. These charge carriers, namely electrons and holes are generated and separated in metal oxides due to their irradiation with light which might target some organic molecules [5]. During the last decade, there has been a tremendous effort dedicated towards using these metal oxides due to their spectacular photostability and removal efficiency of dyes from wastewaters [15, 16, 17]. Nevertheless, their efficiency in removing dyes from wastewater was not that high enough to be largely used. Also, the generation of photo charge carriers is limited by their absorption range [18, 19]. Hence, modification such as doping has been attempted to enhance the photocatalytic activity of the used metal oxide photocatalyst [20, 21, 22, 23]. Doping has been largely adopted in the literature to promote the efficiency of the used photocatalysts and with many different materials, such as transition metals [20] or noble metals [18, 19, 24], semiconductors [25, 26], and reduced graphene oxides [27]. The photocatalytic characteristics of these doped metal oxides have demonstrated an excellent photocatalytic degradation of some dyes in wastewater [28, 29, 30, 31]. Herein, we show the synthesis of NiO:V_2_O_5_ composite nanomaterials via using sol-gel method. Additionally, we show the optimum condition such as pH and temperature to control the structure and size of the synthesized nanoparticles. Thereafter, the synthesis composite nanoparticles with low concentration was used in the degradation of methyl orange in wastewater under irradiation with sun light.

## 2 Materials and methods

All of the chemicals used in this study were used without further purification. Nickel acetate (Ni(CH_3_CO_2_)_2_·2H_2_O), vanadium nitrate NH_4_VO_3_, methanol, absolute ethanol (98.99 %) and acetic acid were sourced from Sigma Aldrich.

The V_2_O_5_ nanomaterials were synthesized using sol-gel method. The particle size and morphologies of the powder were observed using Transmission Electron Microscopy (TEM) and Scanning Electron Microscopy (SEM) respectively. The efficiency of the synthesized photocatalyst was determined by measuring the absorbance of the methyl orange at different irradiation intervals. The absorbance at λmax = 450 nm of the supernatants was determined using ultraviolet visible spectrophotometer by PerkinElmer.

### 2.1 Synthesis of V_2_O_5_ nanomaterials (sol-gel method)

There are several methods for synthesizing metal oxide nanomaterials and one of them is called sol-gel method [18]. In this method, particles are suspended in the solvent and produce sol then it is converted in to gel. To follow this protocol, two samples of 0.6 g of vanadium nitrate (NH_4_VO_3_) was mixed with a solution of 100 mL of absolute ethanol, 100 mL of methanol and several drops of acetic acid to adjust pH to 2.5 and 1.8. Thereafter, the mixtures were stirred with heating at 50 °C for four hours. The resulting solution was evaporated at ambient temperature and calcined for two hours at temperature of 370 °C and 500 °C.

### 2.2 Characterization of V_2_O_5_ nanomaterials

The synthesized V_2_O_5_ nanomaterials were characterized using Scanning Electron Microscope (SEM) and Transmission Electron Microscope (TEM) before and after the compositing.

A 4–7 mg of the V_2_O_5_ nanomaterials was dissolved in 0.5 mL of absolute ethanol and sonicated for 10–15 minutes. A drop of this solution was introduced into a wax film, then a carbon-Cu grid was placed on the top and allowed to be in contact with the drop for 1 minute. The grid was removed, dried with a tissue, and then stored in a box until use for SEM and EDAS SEM characterizations.

### 2.3 Preparation of NiO:V_2_O_5_ composite

In the preparation of NiO- V_2_O_5_ photocatalyst, a (95: 5) % of NiO: V_2_O_5_ was used. The solution (0.4 g nickel acetate with 80 ml of the organic solution) stirred for 2 hours until the solution becomes homogenous. Then nano V_2_O_5_ was added slowly to the solution and stirred using magnetic stirring heater until it became very thick. The catalyst dried at ambient temperature for 24 hours and calcined in the furnace at temperature of 250 °C for 2 hours was performed on the prepared sample.

### 2.4 Preparation of methyl orange (MO) solutions

A stock solution of 1000 mg/L was prepared in ultrapure water, thereafter a set of solutions (30 samples) was prepared by sequential dilution down to 0.02 mg/L was prepared by sequential dilution. The absorption spectra of these solutions were by collected using a UV-Visible spectrophotometer, and the absorbance of them at λ_max_ (450 nm) was measured in order to extract MO calibration curve for further validation.

The photocatalytic activity of NiO:V_2_O_5_ was tested via adding the photocatalyst to three MO solutions of (100 mg/L). All solution were stirred for 10 minutes intervals over the course of 100 minutes. The first solution was stirred in a dark place, the second solution was and stirred under the visible sun light, and the third one was stirred, heated (50 °C), and exposed to visible light up to 70 minutes at the same time intervals as well. To ensure that there is no further change in the color of MO, we collected the absorption spectrum for up to 100 minutes.

The absorbance of these solutions were measured before and after the stirring in dark and under light for comparison.

## 3 Results and discussion

The synthesized V_2_O_5_ nanomaterials were characterized using SEM and TEM microscope.

**Fig. 1:**
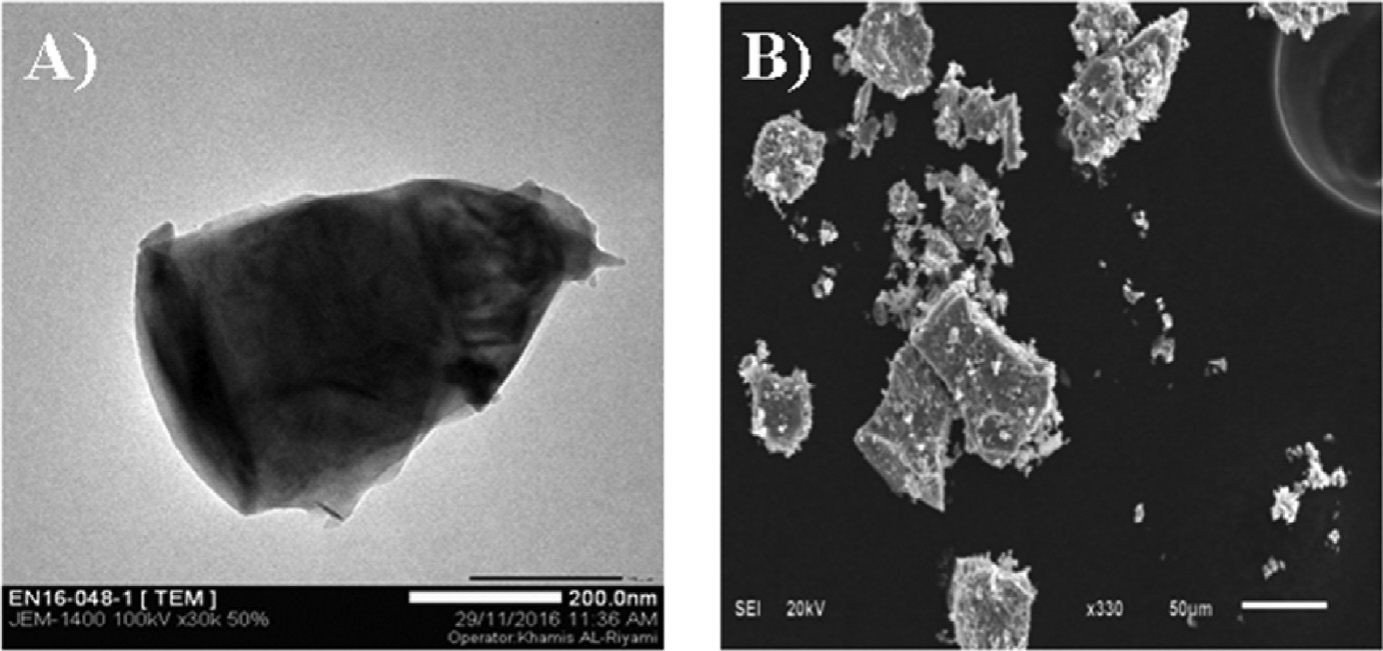
A TEM image of V_2_O_5_ neat, B SEM image of V_2_O_5_ neat both of them synthesized at 500 °C and pH 2.5.

The SEM and TEM images (Fig. 1 A, B) revealed a non-homogenous morphology of the particles at 500 °C and 2.5 PH, in compared to the composite ones which were more crystalline and uniform in size at 500 °C and 1.8 pH. The size of the V_2_O_5_ nanomaterials was around 200 nm. In addition, the optimum conditions for the synthesis of V_2_O_5_ nanomaterials, such as temperature and pH were 500 °C and 1.8 respectively and as depicted in Fig. 1 (A, B).

**Fig. 2:**
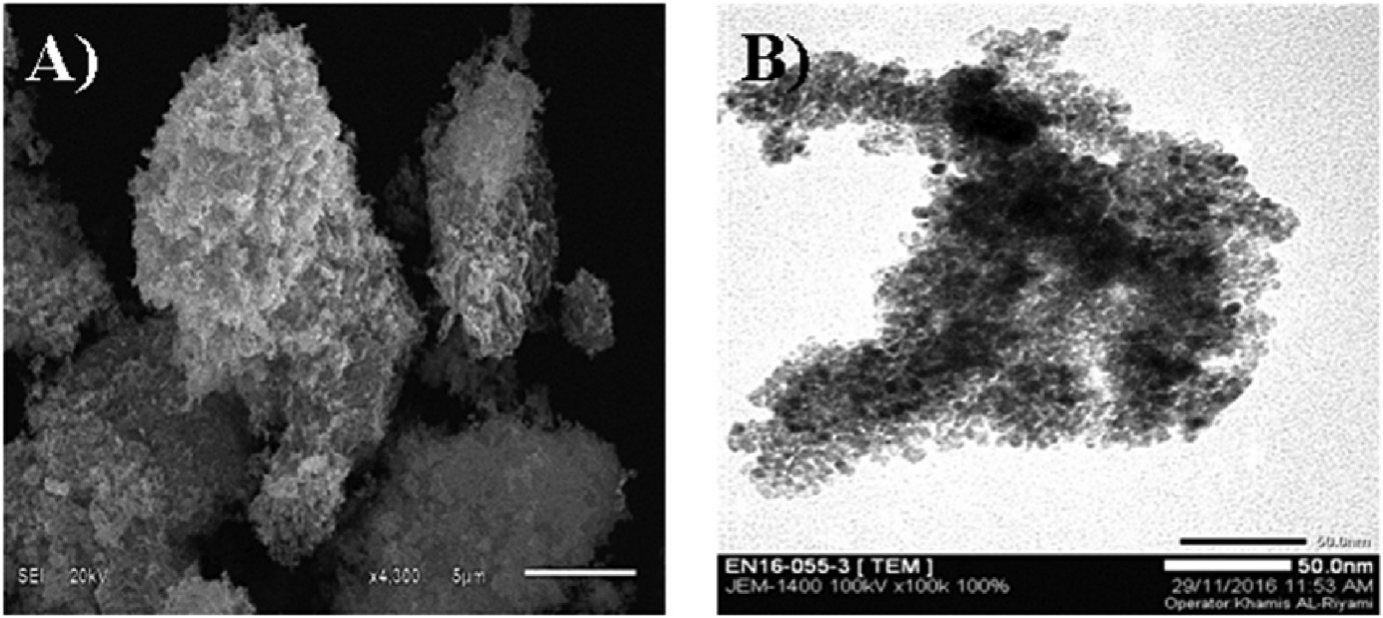
A, and B SEM and TEM images for NiO:V_2_O_5_ composite respectively, 500 °C and pH 1.8.

The synthesized NiO:V_2_O_5_ composite was also characterized by SEM and TEM as shown in Fig. 2 A, and B respectively.

**Fig. 3:**
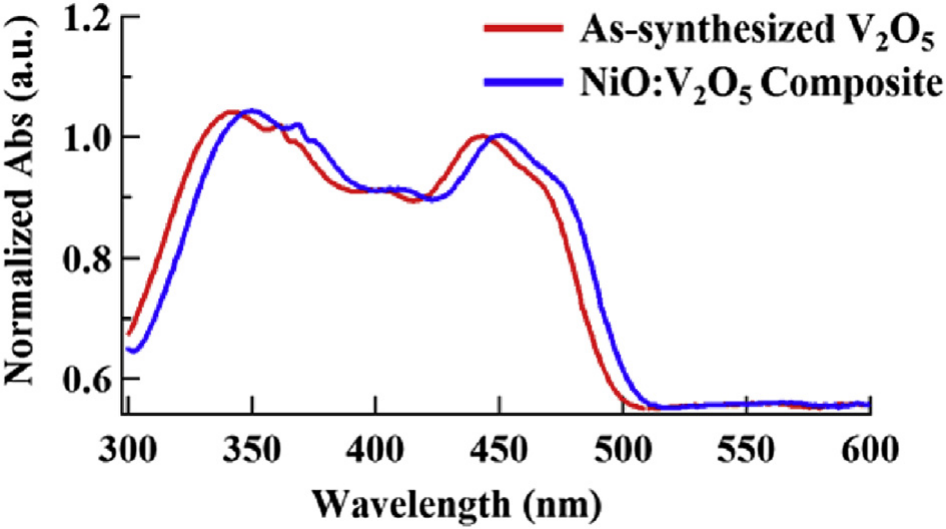
The absorption spectra of the as-synthesized V_2_O_5_ (red trace) and the NiO:V_2_O_5_ composite (blue trace). Both spectra were normalized to unity.

NiO:V_2_O_5_ photocatalyst has a surface area of 94.4 m^2^/g (determined by BET method) reflecting a very high surface area in compared to the reported in the literature [32, 33, 34]. Also, to get a clear picture of the V_2_O_5_ composite photochemical characteristics, a UV-Visible absorption spectra of the as-synthesized V_2_O_5_ and after the introduction of NiO were collected within the range of 300–600 nm as depicted in Fig. 3. The absorption spectra revealed a red shift in the absorption of the V_2_O_5_ composite in compared to the as-synthesized one. The red shift include means larger absorption coefficient for the composite which in turn increase the photocatalytic activity of it within the visible region of the spectrum.

The NiO:V_2_O_5_ composite were employed in the removal of MO dyes from their solutions. A solution of 100 ppm concentration was chosen which underwent a stirring after mixing NiO:V_2_O_5_ for 75 minutes. In fact, there was only a very marginal change in the MO color which means that the removal of methyl orange cannot be achieved if only nanomaterials added to the dye in dark condition scenario. In this case, it was indicated that light is necessary to activate the NiO:V_2_O_5_ to function properly.

**Fig. 4:**
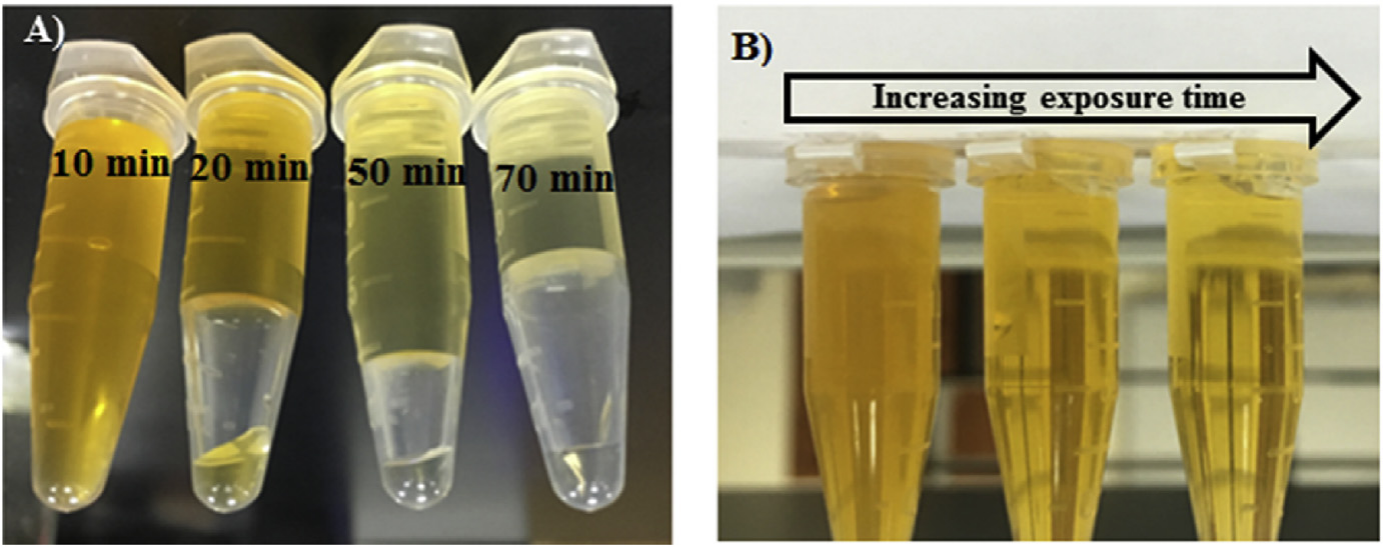
A the MO solution of 100 mg/L after stirring for 70 minutes in a dark place, and B is the 100 mg/L MO solution after stirring for 70 minutes under sun light.

The MO dye solution then was allowed to be mixed with the nanomaterial for the same period of time, but with exposure to visible light. Here we observed a significant reduction of MO absorption as a result of exposing the MO-nanomaterial mixture to the visible light. Hence, the photocatalytic degradation of MO via using NiO:V_2_O_5_ composite nanomaterial was proven to be efficient from the decolorization of MO solution as depicted in Fig. 4, and indicated by the decrease of the absorption at the maximum absorption peak. Samples were collected every 10 minutes for UV test purpose.

**Fig. 5:**
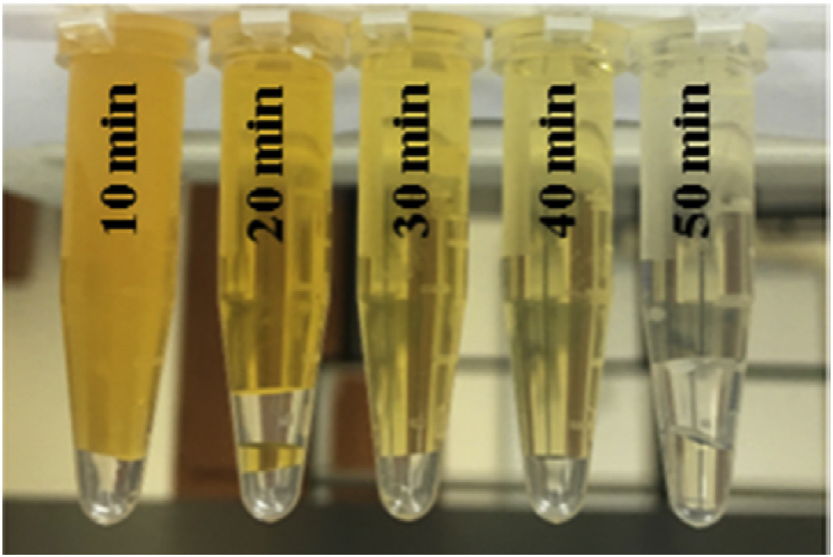
The significant photocatalytic degradation of MO upon increasing temperature to 50 °C.

In a previous report [35], it has been found that imposing high temperatures can lead to significant photocatalytic degradation of a dye from waste water. That is why we heated up the nanomaterial-MO mixture to 50 °C less than 52 minutes and under the exposure to the visible light. It was quite clear that the temperature has a prominent contribution to enhance the degradation of MO as we saw a very high decolorization of MO solution as shown in Fig. 5 with shorter time needed.

**Fig. 6:**
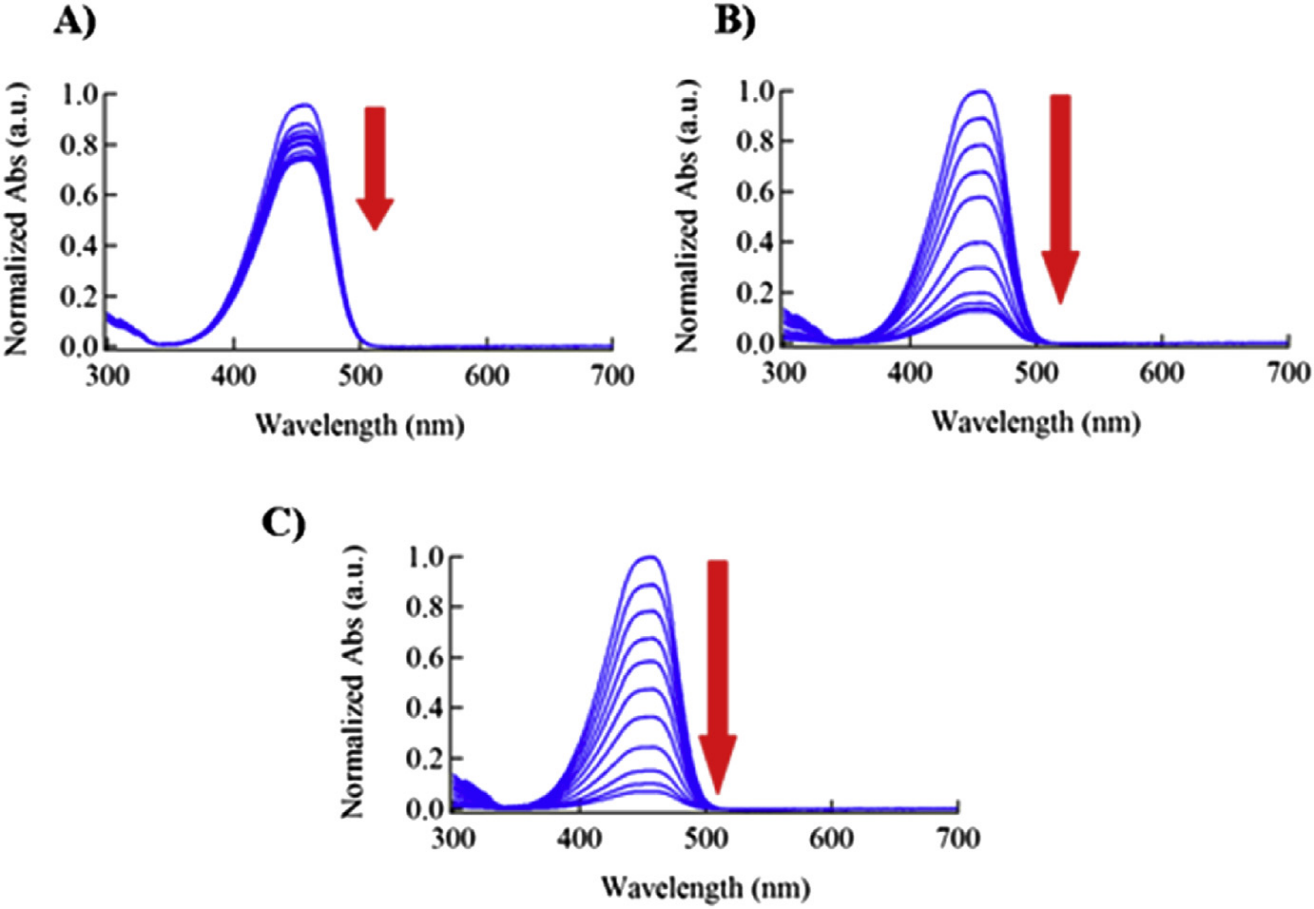
A) The absorption spectrum of MO stirred in the dark. B) The absorption spectrum of MO stirred under sun light, and C) is the absorption spectrum stirred under sun light and heated to 50 °C. Red arrows indicate the reduction of MO absorbance as a result of stirring, light irradiation and heating. All MO solutions were mixed with the composite.

The decolorization effect was monitored via collecting the absorption spectra of the MO dye at 10 minutes time intervals over the course of 100 minutes. These absorption spectra were collected for the samples which were in contact with the Ni:V_2_O_5_ photocatalyst in dark, irradiated by room light at room temperature, and after the heating up to 50 °C. The absorption spectra of MO at these conditions are portrayed in Fig. 6.

**Fig. 7:**
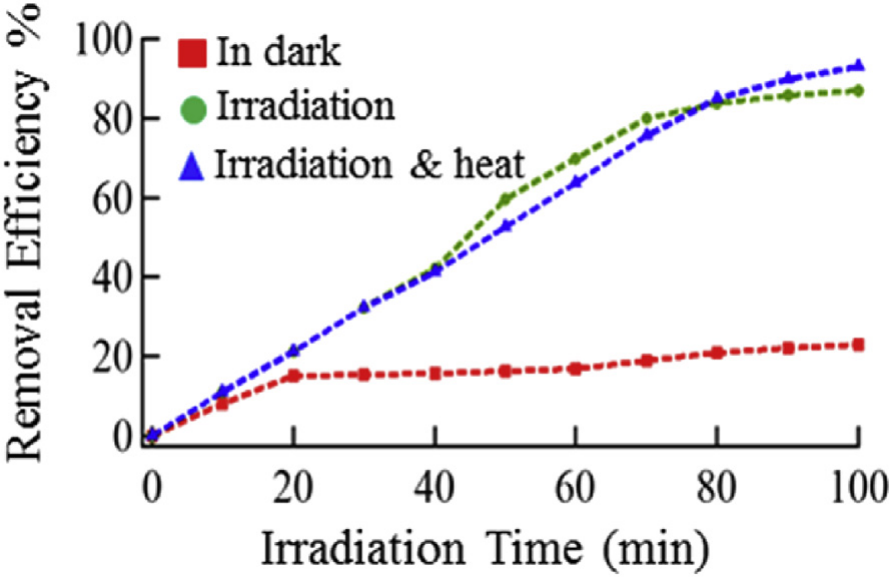
The photocatalytic degradation efficiency of MO at different time intervals. The red squares represent the degradation efficiency of MO in dark. The green circles are the degradation efficiencies upon exposure to room light, and the blue triangles indicate the degradation of MO after irradiation, and heating at 50 °C.

The further exposure to visible light, stirring, and heating did not change the absorbance of MO solution as shown in Fig. 7, which is attributed to the fact that the photocatalyst has a specific number of active sites that the MO can adsorb and being degraded. In this case, the saturation of the NiO:V_2_O_5_ surface prohibited any additional accommodation of MO molecules on the surface. The maximum degraded for the MO were 88% and 93% for conditions B and C respectively. Cetinkaya T et al. [35] have reported that they could achieve up to 40% as photocatalytic degradation of acid orange (AO_7_) dye after 2 hours under the UV radiation. On the other hand, Dnyaneshwar R. et al. [32] result in their comparative study showed 53% as maximum efficiency could be reached for the decolorization for 2 hours as period of reaction and it reached to 95% after 4 hours.

The obtained photocatalytic efficiency of these composites is suggestive of an excellent removal of MO and potentially similar dyes from waste water effluents consequently alleviating one of the sources of pollution in our environment. As benchmark for this experiment, the dyes degrade was achieved within short period of time (around 70 minutes) and low mass catalyst needed (100 mg/L) in compared with previous reports [11, 32, 33, 34].

Another salient feature for this photocatalyst worth mentioning is that the activation of the synthesized V_2_O_5_ composite photocatalyst was enhanced under the irradiation by room light within the ultraviolet and visible region. This broader absorption facilitates the charge carrier generation (electrons and holes) which consequently increase the capacity of entrapping MO molecules on the surface of NiO:V_2_O_5_ particles [36].

## 4 Conclusion

The photocatalytic degradation of the synthesized NiO:V_2_O_5_ nanomaterials was very efficient via controlling several parameters. These nanomaterials were synthesized via optimizing temperature and pH. The NiO:V_2_O_5_ nanomaterials were tested and proved their efficiency which is indicative for using these materials in the removal of other azo dyes from waste water effluents in order to lessen the environmental pollution to aquatic sources.

## Declarations

### Author contribution statement

Fazal Mabood, Hazim Abbas, Sausan Alyaqoobi: Conceived and designed the experiments, Analyzed and interpreted the data.

Lamya Al Amouri: Conceived and designed the experiments, Performed the experiments, Analyzed and interpreted the data.

Salam Mohammed, Emad Yousif and Ali Abd Ali: Conceived and designed the experiments, Analyzed and interpreted the data, Wrote the paper.

### Funding statement

This work was supported by Nizwa University, and Al-Nahrain University.

### Competing interest statement

The authors declare no conflict of interest.

### Additional information

No additional information is available for this paper.
